# A novel model based on necroptosis-related genes for predicting immune status and prognosis in glioma

**DOI:** 10.3389/fimmu.2022.1027794

**Published:** 2022-10-25

**Authors:** Ying-Shi Yuan, Xin Jin, Lu Chen, Jia-Min Liao, Yang Zhang, Ke-Wei Yu, Wei-Kang Li, Shun-Wang Cao, Xian-Zhang Huang, Chun-Min Kang

**Affiliations:** ^1^ Department of Laboratory Medicine, The Second Clinical College of Guangzhou University of Chinese Medicine, Guangzhou, Guangdong, China; ^2^ Department of Laboratory Medicine, Guangdong Provincial Hospital of Chinese Medicine, Guangzhou, Guangdong, China; ^3^ Department of Neurosurgery, Guangdong 999 Brain Hospital, Guangzhou, Guangdong, China

**Keywords:** glioma, necroptosis, prognostic model, immune infiltration, immune checkpoint

## Abstract

**Background:**

Glioma is a highly aggressive brain cancer with a poor prognosis. Necroptosis is a form of programmed cell death occurring during tumor development and in immune microenvironments. The prognostic value of necroptosis in glioma is unclear. This study aimed to develop a prognostic glioma model based on necroptosis.

**Methods:**

A necroptosis-related risk model was constructed by Cox regression analysis based on The Cancer Genome Atlas (TCGA) training set, validated in two Chinese Glioma Genome Atlas (CGGA) validation sets. We explored the differences in immune infiltration and immune checkpoint genes between low and high risk groups and constructed a nomogram. Moreover, we compiled a third validation cohort including 43 glioma patients. The expression of necroptosis-related genes was verified in matched tissues using immunochemical staining in the third cohort, and we analyzed their relationship to clinicopathological features.

**Results:**

Three necroptosis-related differentially expressed genes (*EZH2*, *LEF1*, and *CASP1*) were selected to construct the prognostic model. Glioma patients with a high risk score in the TCGA and CGGA cohorts had significantly shorter overall survival. The necroptosis-related risk model and nomogram exhibited good predictive performance in the TCGA training set and the CGGA validation sets. Furthermore, patients in the high risk group had higher immune infiltration status and higher expression of immune checkpoint genes, which was positively correlated with poorer outcomes. In the third validation cohort, the expression levels of the three proteins encoded by *EZH2*, *LEF1*, and *CASP1* in glioma tissues were significantly higher than those from paracancerous tissues. They were also closely associated with disease severity and prognosis.

**Conclusions:**

Our necroptosis-related risk model can be used to predict the prognosis of glioma patients and improve prognostic accuracy, which may provide potential therapeutic targets and a theoretical basis for treatment.

## Introduction

Glioma is the most common and lethal primary intracranial malignancy in the central nervous system. It is histologically divided into low grade (LGG, WHO grade I/II) and high grade (HGG, WHO grade III/IV) (1). The median survival time of LGG patients is 10−15 years, while nearly 70% of LGG cases transform into HGG (2, 3). Glioblastoma (GBM) is the most aggressive form, with the worst 5-year survival rate of 6.8% (4), and it accounts for 60% of all HGG cases (5). The standard treatment for glioma is surgical resection combined with radiotherapy and chemotherapy. However, most gliomas recur or progress, and the long-term effect of treatments is limited due to their highly invasive and infiltrative features (4, 6).

Necroptosis is a form of programmed cell death characterized by rupture of the cellular membrane, swelling of organelles, cytolysis, and a robust inflammatory response (7). Necroptosis plays an important role in the regulation of tumor microenvironment (7, 8), whereas immunotherapy is a promising therapeutic strategy in glioma (9, 10). It creates an inflammatory immune microenvironment with either anti-tumor or pro-tumor effects. Necroptosis induces a strong adaptive immune response to exert anti-tumor activity (7). In the latter scenario, necroptotic cancer cells not only attract myeloid-derived suppressor cells and/or tumor-associated macrophages and cause tumor-associated immune suppression but also release cytokines that promote angiogenesis, cancer proliferation, and metastasis (11).Necroptosis is considered to be a promising strategy in treatment evaluation of a broad spectrum of tumors (7, 12). Pagano et al. reported that N6-isopentenyladenosine could bypass the apoptosis resistance mechanism and induce necroptosis in GBM cells thereby offering higher therapeutic efficacy (13). However, the exact role of necroptosis in glioma has remained relatively under-researched. A search for specific biomarkers based on necroptosis hallmark might be more precise and effective in glioma management, which could shed new light on immunotherapy and prognosis. Recently, several clinically significant biomarkers, such as isocitrate dehydrogenase (IDH) mutation, chromosome 1p19q codeletion, and TERT promoter mutations, have been identified in glioma that may open specific therapeutic options and improve prognostic assessment. However, personal disease management targeted to these biomarkers has yet to achieve a breakthrough, and a substantial proportion of patients face a dismal prognosis (14). Although numerous prognostic models for glioma have been developed, most of them contain more genes. Real clinical samples validation was usually confined in the level of gene expression, rather than the prognostic model, which actually do not have enough persuasion (15–19).

In this study, a prognostic model including three necroptosis-related genes (*EZH2*, Enhancer of zeste homolog 2; *LEF1*, Lymphocyte enhancer factor-1, and *CASP1*, Caspase-1) was constructed based on The Cancer Genome Atlas (TCGA) training set and validated in two Chinese Glioma Genome Atlas (CGGA) validation sets. Moreover, we constructed a prognostic nomogram for clinical application and conducted immune-related analysis in glioma patients. To further validate the reliability of the necroptosis-related risk model, we compiled a third validation cohort including 43 glioma patients. The protein expression of EZH2, LEF1, and CASP1 were determined by immunochemistry (IHC) staining in the third cohort, and their association with clinicopathologic features was analyzed. Our study suggested a potential role of necroptosis-related genes in glioma, which might provide a new perspective on prognosis and treatment selection for glioma.

## Materials and methods

### Datasets and processing

Patient data were downloaded from the NCBI Gene Expression Omnibus (GEO, https://www.ncbi.nlm.nih.gov/gds/ ), TCGA (https://portal.gdc.cancer.gov/ ), and CGGA (http://www.cgga.org.cn ). The GSE66354 dataset was generated using the GPL570 [HG-U133_Plus_2] Affymetrix Human Genome U133 Plus 2.0 Array platform. For the TCGA cohort, the counts value matrix was for differential analysis, while the TPM value matrix was for the other analyses. mRNA expression data from the CGGA301 and CGGA325 cohorts was generated with the Agilent Whole Human Genome Array and the Illumina HiSeq 2000 sequencing system, respectively. Data from GSE66354, TCGA, CGGA datasets were normalized respectively. The clinical information of the glioma patients in the TCGA and CGGA cohorts is summarized in **Supplementary Table 1**. Patients with incomplete prognostic details were excluded from the risk model.

### Patients

The third cohort comprised 43 glioma patients and was investigated to validate the necroptosis-related risk model (**Supplementary Table 2**). Patients were diagnosed with glioma between 2017 and 2022, with follow-up through July, 2022. Median follow-up time was 1098 days (IQR 491–1824 days). This study was approved by the Ethics Committee of Guangdong 999 Brain Hospital (Approval number 2021-01-084), and written informed consent was obtained from all participants.

### Differential expression and functional enrichment

The GSE66354 dataset contained 13 non-tumor samples and 136 brain tumor samples, while the TCGA cohort contained 169 GBM, 529 LGG, and five non-tumor samples. The differentially expressed genes (DEGs) between the glioma and non-tumor samples in the TCGA cohort and the GSE66354 dataset were identified using the DESeq2 R package (DESeq2 v.1.26.0) and the limma package, respectively. Threshold values were set as adjusted *p* < 0.05 and absolute logFC value > 1. A Venn diagram was used to select the overlapping DEGs. In addition, 261 necroptosis-related genes were obtained from previous studies, GeneCards (with a correlation score > 1.0, https://www.genecards.org/), the Gene Set Enrichment Analysis (GSEA, http://www.gsea-msigdb.org/gsea/index.jsp ), the Kyoto Encyclopedia of Genes and Genomes database (KEGG, https://www.kegg.jp/ ) by searching “necroptosis” (**Supplementary Table 3**). Functional enrichment analysis for the intersection of DEGs and necroptosis-related genes, including gene ontology (GO) analysis and KEGG analysis, was performed using the cluster profiler R package. Threshold values were set as an adjusted *p* < 0.05.

### Necroptosis-related risk model

For necroptosis-related DEGs, a protein-protein interaction (PPI) network was constructed by STRING and Cytoscape software. The top 10 hub genes ranked by the degree algorithm were selected for univariate and multivariate Cox regression analyses. Genes with a *p*-value < 0.05 were considered to have significant prognostic value. All survival analyses were performed using the survival package (version 3.2-10). Subsequently, the optimal prognosis-related genes were identified to construct a necroptosis-related risk model for predicting overall survival (OS) with the following formula: risk score = expression level of gene_1_ × β_1_ + expression level of gene_2_ × β_2_ +…+ expression level of gene_n_ × β_n_; in which β is the regression coefficient calculated by multivariate Cox regression. Based on the median risk score, patients were divided into high and low risk groups. Kaplan-Meier (K-M) survival analysis was performed to estimate the association between the risk groups and OS. The accuracy of the necroptosis-related risk model in predicting patient outcomes was evaluated by receiver operating characteristic (ROC) curves using the timeROC R package.

### Prognostic nomogram

To evaluate whether the necroptosis-related risk model can serve as an independent prognostic factor, univariate and multivariate Cox proportional hazards regression analyses were performed with clinicopathologic features, including age, sex, WHO grade, IDH mutation status, and 1p19q codeletion status, radiotherapy and chemotherapy. Nomogram was constructed with the rms R package according to Cox proportional hazard test (**Supplementary Figure 1**) and multivariate Cox regression (**Supplementary Figure 2** and **Supplementary Table 4**). Calibration curves, ROC curves, and the concordance index (C-index) were used to evaluate the predictive performance of the nomogram. The prognostic nomogram was externally validated in the CGGA301 and CGGA325 cohorts.

### Immune infiltration

In the TCGA training set, ssGSEA and ESTIMATE were applied to evaluate the immune infiltration status in each glioma patient using the GSVA and estimate packages, respectively. Twenty-four specific immune cell subsets were obtained based on the ssGSEA algorithm (20). The immune infiltration level was compared between patients from low and high risk groups with the Wilcoxon signed-rank test. Spearman’s correlation analysis of risk score, immune score, stromal score, and ESTIMATE score was also conducted.

### Immunohistochemistry

To determine the expression of three necroptosis-related genes between glioma and paracancerous tissues, 43 paired samples from Guangdong 999 Brain Hospital between 2017 and 2022 were collected for immunohistochemical (IHC) staining. The following antibodies were used: EZH2 (Abcam, ab283270), LEF1 (Abcam, ab137872), and CASP1 (Cell Signaling Technology, #98033). IHC-stained sections were imaged with a Leica microscope. For IHC quantification, the average percentage of positive cells in five random fields was calculated by Fiji.

### Statistical analysis

Bioinformatics analysis was conducted using R software (version 3.6.3). The statistical difference of two groups was compared through the Wilcox test, significance difference of three groups was tested with Kruskal-Wallis test. The proportional hazard test in the Cox models was assessed with the log-log cumulative survival graph, Kaplan-Meier survival analysis and Schoenfeld’ test using SPSS20.0 before the construction of nomogram (**Supplementary Figure 1**). Logarithmic fit was made with the software Origin 2021. Spearman’s rank test was used for correlation analysis. Hazard ratios (HRs) and 95% confidence intervals (CIs) are reported where applicable. A *p*-value of < 0.05 was considered statistically significant (^*^ *p* < 0.05; ^**^ *p* < 0.01; ^***^ *p* < 0.001; ns, not significant).

## Results

### Necroptosis-related DEGs in glioma patients and enrichment analysis

**Figure 1:**
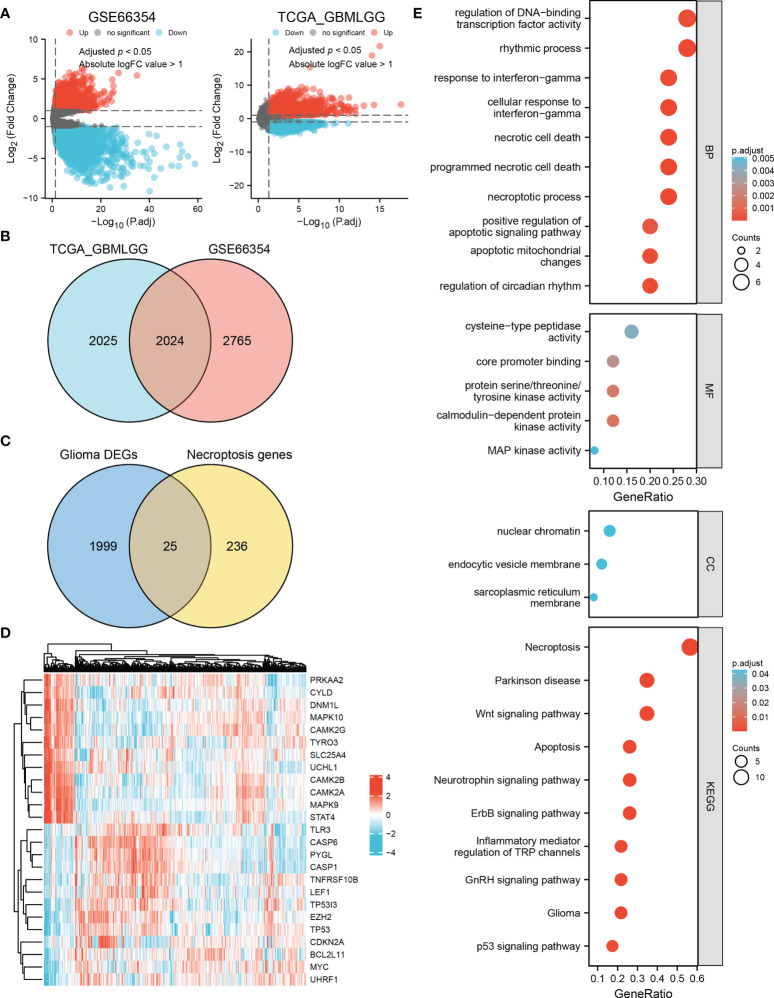
Identification of necroptosis-related DEGs in glioma and enrichment analysis. **(A)** Volcano plots of DEGs in the TCGA and GSE66354 datasets. Red indicates upregulated genes, and blue indicates downregulated genes. Threshold values were set as adjust *p* < 0.05 and absolute logFC value > 1. **(B)** 2024 differential genes in the intersection of these two datasets. **(C)** Venn diagram showing 25 necroptosis-related DEGs in glioma. **(D)** Heat map of the expression level of the 25 necroptosis-related DEGs in the TCGA dataset. **(E)** Biological process (BP), molecular function (MF), cellular component (CC), and Kyoto Encyclopedia of Genes and Genomes (KEGG) pathways are enriched for the 25 necroptosis-related DEGs.

By analyzing the TCGA and GSE66354 datasets, 2024 DEGs were identified between glioma and non-tumor samples (**Figures 1A, B**). According to the Venn analysis, 25 necroptosis-related DEGs (the intersection of the 2024 DEGs and 261 necroptosis-related genes, **Figure 1C**) were selected for further analysis, among which 13 were upregulated and 12 were downregulated (**Figure 1D** and **Supplementary Table 5**). Enrichment analyses revealed that these genes are significantly enriched in the necroptotic process, programmed necrotic cell death, cellular response to interferon-gamma, and positive regulation of the apoptotic signaling pathway. KEGG analysis showed that necroptosis, the Wnt signaling pathway, the ErbB signaling pathway, the neurotrophin signaling pathway, apoptosis, glioma, and the p53 signaling pathway were enriched (**Figure 1E** and **Supplementary Table 6**).

### Necroptosis-related prognostic DEGs

**Figure 2:**
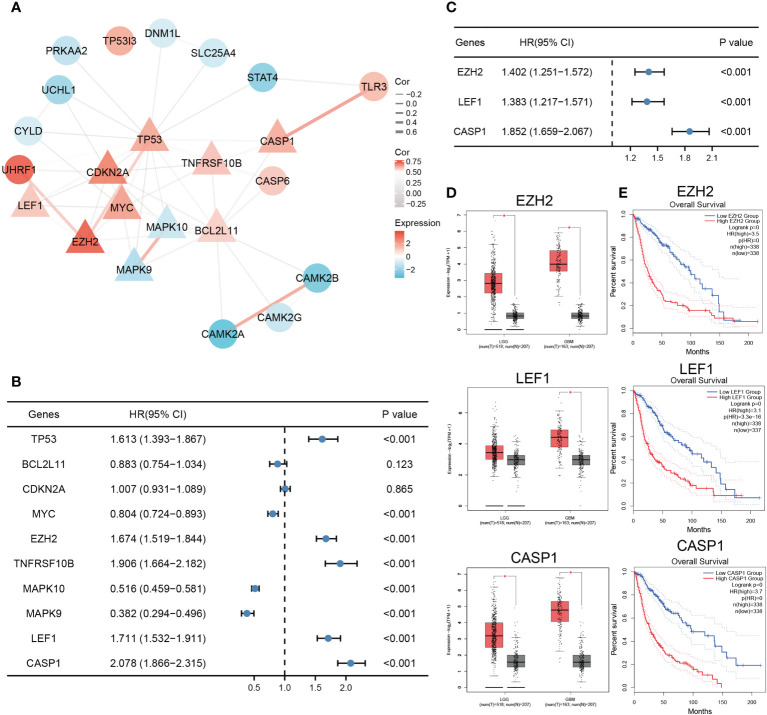
Identification of necroptosis-related prognostic DEGs. **(A)** The PPI network of the necroptosis-related DEGs. Triangles represent hub genes identified by the degree algorithm. The thickness of the line represents the strength of the correlation. **(B)** Univariate Cox regression analysis of top ten hub genes. **(C)** *EZH2*, *LEF1*, and *CASP1* were identified as significant prognostic genes for glioma in multivariate Cox regression analysis. **(D, E)** The expression and survival analysis of *EZH2*, *LEF1*, and *CASP1* in glioma based on GEPIA (^*^ *p* < 0.05).

Analysis of the 25 necroptosis-related DEGs using STRING revealed the PPI network. The result was then visualized with Cytoscape, and the top 10 hub genes were selected for further analysis (**Figure 2A**). By performing univariate Cox regression in the TCGA training set, eight genes (*TP53*, *MYC*, *EZH2*, *TNFRSF10B*, *MAPK10*, *MAPK9*, *LEF1*, and *CASP1*) were identified as potential risk factors related to OS (**Figure 2B**). Subsequent multivariate Cox regression analysis indicated that three genes, *EZH2*, *LEF1*, and *CASP1*, exhibited significant prognostic value for glioma (**Figure 2C**). Based on Gene Expression Profiling Interactive Analysis (GEPIA), the expression levels of these genes were significantly higher in glioma patients, especially in GBM patients (**Figure 2D**). K-M analysis indicated that the high expression groups had poorer OS than the low expression groups (**Figure 2E**).

### Necroptosis-related risk model

**Figure 3:**
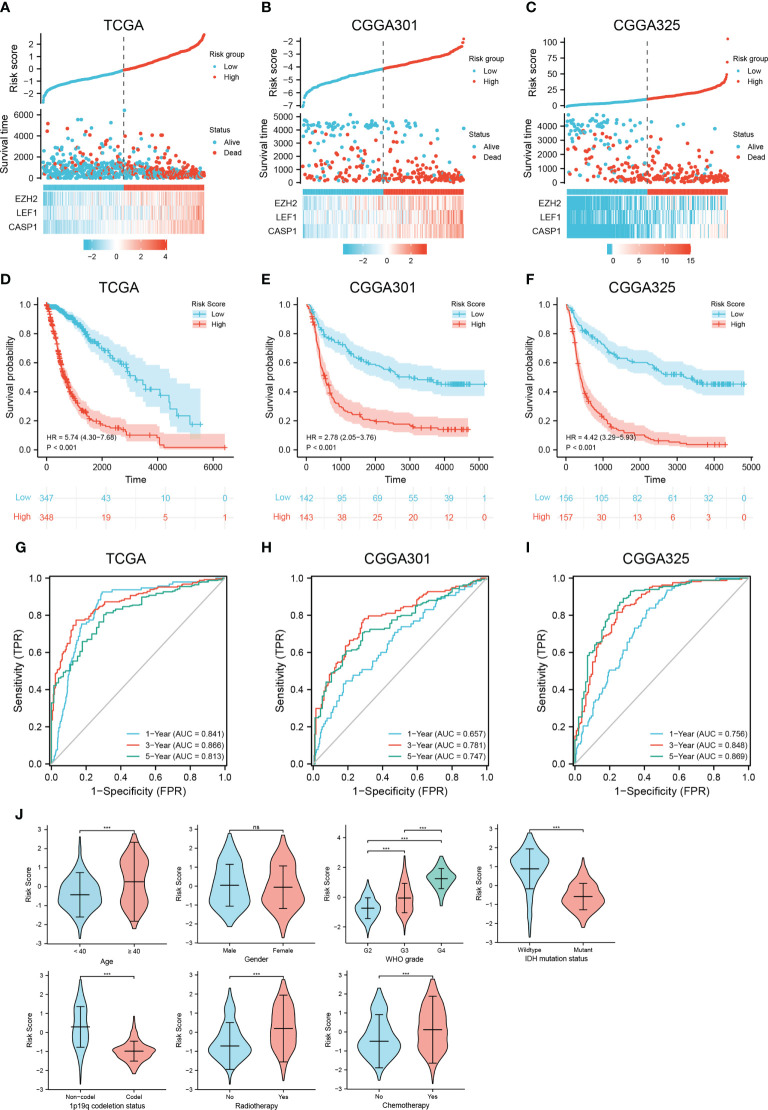
Construction and validation of a necroptosis-related risk model and its association with clinicopathologic features. **(A–C)** The risk score, survival distributions, and expression heatmaps of *EZH2*, *LEF1*, and *CASP1* in the TCGA and CGGA cohorts. **(D–F)** Kaplan-Meier survival analysis based on the necroptosis-related risk model in the TCGA and CGGA cohorts. **(G–I)** ROC curves predicting 1-, 3-, 5-year OS of glioma patients in the TCGA and CGGA cohorts. **(J)** The associations between the necroptosis-related risk model and clinicopathological features (^***^ *p* < 0.001; ns, not significant).

The necroptosis-related risk model was constructed and calculated as follows: risk score = EZH2 * 0.338 + LEF1 * 0.324 + CASP1 * 0.616 - 4.300. Median risk scores were used to classify patients into low and high risk groups. Patients in the high risk group had higher expression of the prognostic genes and poorer outcomes (**Figures 3A–C**). In the TCGA training set, K-M analysis confirmed that patients with high risk scores had significantly poorer OS than those with low risk scores (HR=5.74, 95% CI=4.30−7.68, *p* < 0.001). The median survival times of the high- and low risk groups were 8.77 (95% CI, 7.29−12.18) and 1.78 (95% CI, 1.51−2.16) years, respectively (**Figure 3D**). Similar results were found in the CGGA301 and CGGA325 validation sets (CGGA301, HR=2.78, 95% CI=2.05−3.76; CGGA325, HR=4.42, 95% CI=3.29–5.93; *p* < 0.001; **Figures 3E, F**). To evaluate the specificity and sensitivity of the prognostic risk model in glioma patients, a time-independent ROC curve analysis was conducted. The area under the curve (AUC) of the TCGA training set and the CGGA301 and CGGA325 validation sets were 0.841, 0.657, and 0.756 at 1 year, 0.866, 0.781, and 0.848 at 3 years, and 0.813, 0.747, and 0.869 at 5 years (**Figures 3G–I**), respectively, implying that the necroptosis-related risk model had a good performance in predicting the prognosis of glioma patients. In addition, clinicopathological characteristics were compared between the low and high risk groups. The high risk group of three cohorts was significantly associated with older age, higher WHO grade, IDH wildtype status, 1p19q non-codeletion, and the risk scores were not associated with sex (**Figure 3J** and **Supplementary Figure 3**). Interestingly, in the TCGA cohort, patients with radiotherapy and chemotherapy have higher risk scores compared to those receiving no corresponding treatment, one important reason for this is that patients receiving chemoradiotherapy or radiotherapy were mostly older than 40 years, with high WHO grade and 1p19q codeletion status, which were risk factors of the prognosis in glioma (**Supplementary Table 7**).

### Prognostic nomogram

**Figure 4:**
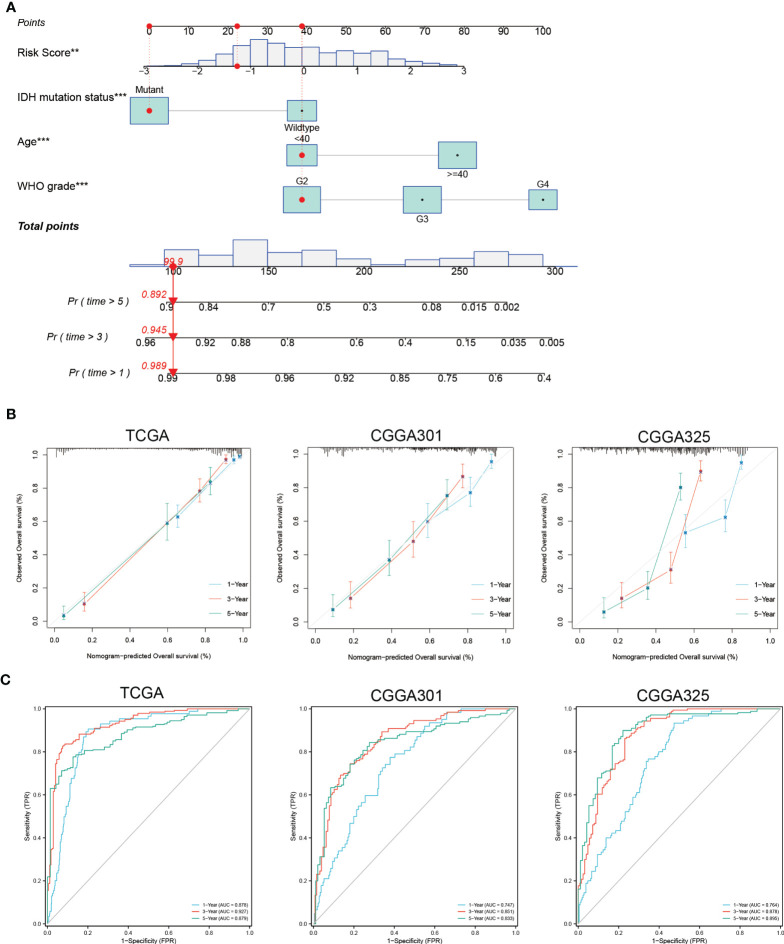
Construction and validation of nomogram based on independent prognostic factors of glioma. **(A)** Prognostic nomogram to predict the 1-, 3-, and 5-year OS of glioma patients. Red dots represent one case from the TCGA training set. **(B)** The 1-, 3-, and 5-year calibration curves for the nomogram in the TCGA and CGGA cohorts. The x-axis and y-axis represent nomogram predicted and actual survival, respectively. The 45-degree gray line represents perfect calibration. **(C)** The 1-, 3-, and 5-year ROC curves for the nomogram in the TCGA and CGGA cohorts. **p < 0.01; ***p < 0.001

When patients were stratified by clinicopathologic features, the necroptosis-related risk score remained an independent prognostic factor whether in the training or validation sets (*p* < 0.05, **Supplementary Table 4**). Multivariate Cox regression showed that age, WHO grade, IDH mutation status, radiotherapy, chemotherapy and risk score were independent prognostic factors (*p* < 0.05). Prognostic factors met the proportional hazard test were integrated into the nomogram model for predicting the 1-, 3-, and 5-year OS of glioma patients (**Figure 4A** and **Supplementary Figures 1**, **2**). The calibration curves showed good consistency between the nomogram prediction and actual probability (**Figure 4B**). The nomogram showed an excellent predictive ability for the 1-, 3- and 5-year OS, with AUCs of 0.878, 0.927 and 0.879, respectively (**Figure 4C**). The C-index of the nomogram was 0.843 (0.832-0.855), which was higher than that of age, WHO grade, IDH mutation status, and necroptosis-related risk score alone (**Supplementary Table 8**). The predictive reliability of the nomogram was well validated in the CGGA301 and CGGA325 cohorts (**Figures 4B, C**), indicating that it might be an effective tool for predicting the prognosis of glioma patients in clinical practice.

### Immune infiltration and immune checkpoint genes

**Figure 5:**
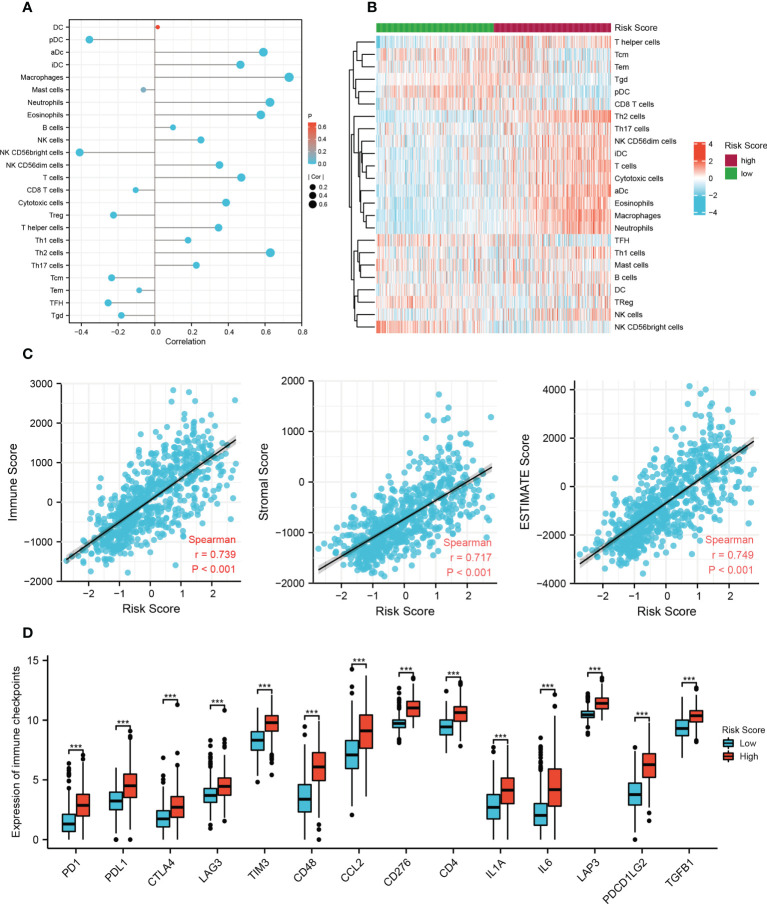
Immune-related analysis of the risk model in the TCGA cohort. **(A)** The correlation between 24 immune cell types and risk score based on the ssGSEA algorithm. DC, dendritic cell; pDC, plasmacytoid dendritic cells; aDC, activated DC; iDC, immature DC; Treg, regulatory T cells; Tcm, T central memory cells; Tem, T effector memory cells; TFH, T follicular helper cells; Tgd, gamma delta T cells. **(B)** Heat map of ssGSEA scores of 24 immune cell types between the low- and high-risk groups. **(C)** The correlation between immune scores and risk scores based on the ESTIMATE algorithm. **(D)** The difference in 14 checkpoint genes expression between the low and high risk groups (^***^ *p* < 0.001).

To provide a reference for clinical immunotherapy, the immune infiltration levels and the expression of immune checkpoint genes among the low and high risk groups were compared based on the TCGA training set. The infiltration scores calculated by ssGSEA showed that there were more infiltrating immune cells, including macrophages, neutrophils, eosinophils, T cells, B cells, and NK cells, in the high-risk group (*p* < 0.01), and most of the correlation coefficients were between 0.35 and 0.75 (**Figures 5A, B**). The high risk scores were positively related to immune, stromal, and ESTIMAT scores, with Spearman correlation coefficients of 0.739, 0.717, and 0.749, respectively (**Figure 5C**). In addition, 14 immune checkpoint genes (*PD1*, *PDL1*, *CTLA4*, *LAG3*, *TIM3*, *CD48*, *CCL2*, *CD276*, *CD4*, *IL1A*, *IL6*, *LAP3*, *PDCD1LG2*, and *TGFB1*) had significantly higher expression in the high-risk group than in the low-risk group (**Figure 5D**), which is consistent with other findings (21, 22). Our results revealed differences in the immune microenvironment between low- and high-risk patients. Glioma patients with high risk scores might benefit from immune checkpoint inhibitor therapy.

### Relationship between the necroptosis-related risk model and clinicopathological features in an independent cohort

**Figure 6:**
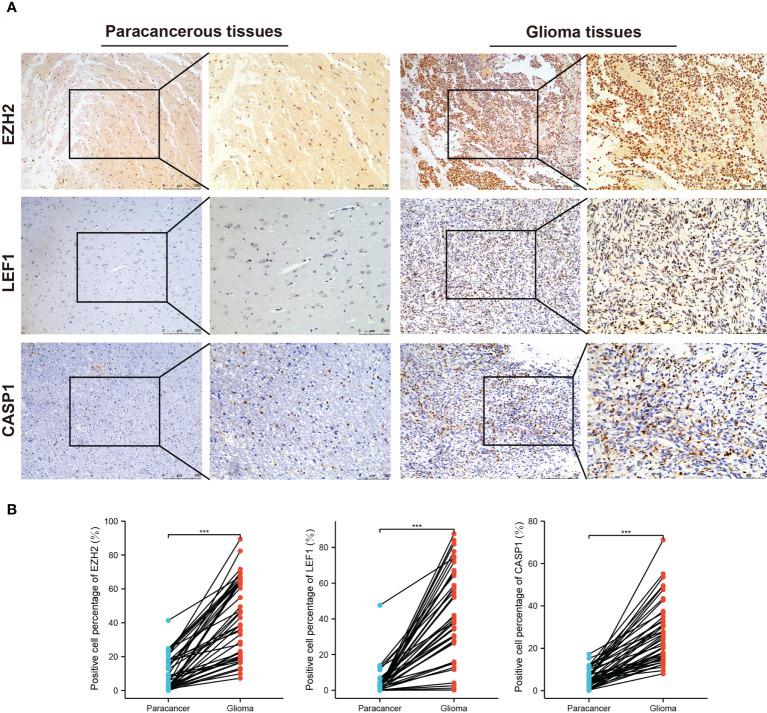
IHC staining for EZH2, LEF1, and CASP1 in the third cohort. **(A)** Representative images of IHC staining in glioma and matched paracancerous samples. The brown color indicates positive staining. **(B)** Elevated EZH2, LEF1, and CASP1 expression levels in 43 glioma tissues. (^***^ *p* < 0.001).

**Figure 7:**
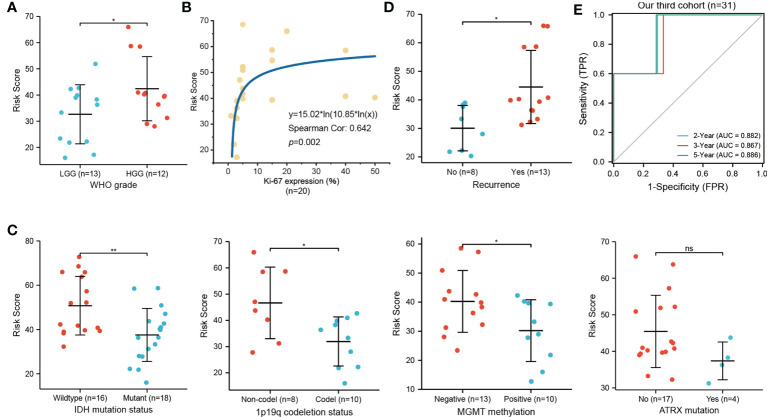
Relationship of necroptosis-related risk score and clinicopathological features in the third cohort. **(A)** HGG patients show a higher risk score than LGG patients (n=25). **(B)** Positive correlation between the Ki-67 expression and risk score (n=20). **(C)** High risk score was significantly related to IDH wildtype (n=34), 1p19q non-codeletion (n=18), and MGMT unmethylated (n=23) status but not to ATRX mutation (n=21). **(D)** Glioma patients with postoperative recurrence show higher risk scores (n=21; ^*^ *p* < 0.05; ^**^ *p* < 0.01; ns, not significant). **(E)** ROC curve for predicting 2-, 3-, 5-year OS of glioma patients in our third cohort (n=31, 2 deaths and 29 alive).

We employed IHC to validate the protein expression of EZH2, LEF1, and CASP1 in 43 glioma tissues and matched paracancerous tissues. In paracancerous tissues, these proteins were only weakly expressed or not expressed. Statistical analysis showed that these three proteins were significantly higher in glioma tissues (**Figures 6A, B**). According to the necroptosis-related risk model, the risk scores of each patient were calculated based on the IHC quantitative results (average percentage of positive cells). In our cohort, the necroptosis-related risk scores were associated with disease severity and prognosis, consistent with the above results. Patients with HGG had a higher risk score than those with LGG (**Figure 7A**). The high risk scores were positively related to the classic indicator of tumor cell proliferation, Ki-67, with a Spearman’s correlation coefficient of 0.642 (**Figure 7B**). The necroptosis-related risk model was also associated with prognostic glioma markers. Patients with IDH mutation, 1p19q codeletion, and MGMT methylation status had lower risk scores (**Figure 7C**). Furthermore, high risk scores were significantly associated with postoperative recurrence (**Figure 7D**). During follow-up, we observed 2 deaths and 29 alive among 43 cases of glioma. The limited number of positive cases makes the ROC curve jagged. However, the AUCs were 0.882, 0.867 and 0.886 at 2-, 3- and 5- year, respectively (**Figure 7E**). In the further study we will continue to follow-up patients and collect the survival data.

## Discussion

In the last decade, despite efforts to explore novel therapeutic strategies, the clinical prognosis of glioma remains dismal. Immunotherapy, which aims to improve antitumor immune responses, becomes the promising therapeutic strategy of glioma treatment (9). Necroptosis is a novel form of programmed cell death that plays an important role in tumor prognosis and regulation of immune microenvironment (7). However, the role of necroptosis in glioma remains to be elucidated. A search for specific biomarkers based on necroptosis hallmark might have clinical value in guiding immunotherapy and prognosis of glioma for follow-up study.

Currently, numerous prognostic models based on specific hallmark have been developed in glioma, but most of them contain more genes and lack of validation of clinical samples. Real clinical samples validation was usually confined in the level of gene expression, rather than the prognostic model, which actually do not have enough persuasion (15–19). In this study, we constructed a necroptosis-related prognostic model that comprised of three genes (*EZH2*, *LEF1* and *CASP1*). The performance of our prognostic model was compared with partially existing risk models using respective formulas in CGGA301 cohort (**Supplementary Figure 5**). AUCs and C-index showed that our three-gene prognostic model achieved similarly great predictive performance compared with existing models (16–18). Despite the presence of higher accuracy in other models, their models contained more genes and lack of samples validation, which restricted the clinical applications (15, 19). Most importantly, our model was well validated in clinical samples. We collected 43 paired glioma tissues and compiled a third validation cohort. We confirmed that the model was associated with clinical outcome as well as some classical biomarkers, including Ki-67, IDH mutation, 1p19q codeletion, MGMT methylation. The clinical sample validation in our third cohort preliminary demonstrated the usability of this prognostic model in clinical applications. We attempted to develop a universal prognostic model by integration analysis of LGG and GBM. However, our prognostic model had similar prediction effect on LGG, GBM and gliomas. A high risk score in both LGG and GBM groups correlated with a poor prognosis and high immune status, consistent with the integration analysis of gliomas (**Supplementary Figure 4**). In summary, our prognostic model based on necroptosis may be more persuasive and has great clinical applications.

Infiltrating immune cells involve in tumorigenesis and tumor progression, not only as prognostic predictors, but also as therapeutic targets and potential biomarkers of response to tumor immunotherapy (23, 24). Therefore, understanding the immune microenvironmental status and the proportion of immune cell infiltration may help optimize treatment strategies and assess prognosis of tumor. In this study, a higher complexity of immune cells was showed in high risk group, and the risk score was significantly positively correlated with the abundance of macrophages and neutrophils. Macrophages are the main immune cells in the brain tumors, have been implicated in tumor proliferation, survival, migration and resistance to anti-angiogenic therapies (25). Increased neutrophil infiltration into tumors is significantly correlated with glioma grade and in glioblastoma with acquired resistance to anti-VEGF therapy, which may have prognostic value in glioma (26). Immunotherapy targeting macrophages or neutrophils might have certain potential in treatment of high risk group. Furthermore, high-risk patients had significantly higher expression of immune checkpoint genes than low-risk patients, and might benefit more from immune checkpoint inhibitor therapy. These findings indicated that necroptosis-related genes may predict or influence immunotherapeutic effects in patients with glioma.

The dysregulation of three necroptosis-related genes (*EZH2*, *LEF1*, and *CASP1*) was significantly related to the OS of glioma patients, especially those with older age, higher WHO grade, wildtype IDH status, and 1p19q codeletion status. EZH2, a histone methyl transferase subunit of a polycomb repressor complex, is associated with inflammatory diseases and numerous cancers (27, 28). Inhibition of EZH2 promotes IFNγ/TNFα-stimulated necroptosis and induces the death of keratinocytes infected by high-risk human papillomavirus (29). The expression of LINC00963 induced by EZH2 signaling could prevent apoptosis in glioma cells (30). Wang showed that EZH2 promotes aerobic glycolysis and increases the growth in glioma cells *via* β-catenin signaling (31). LEF1, a major transcription factor of the Wnt pathway, has been reported to play a significant role in cancer progression, such as in T lymphocyte acute lymphoblastic leukemia, hepatocellular carcinoma, and glioma (32–35). Liu reported that LEF1 is a key regulator of the necroptotic machinery, and knocking it down sensitized chronic lymphocytic leukemia cells to TNFα/zVAD-induced necroptosis (36). Downregulation of LEF1 inhibits cell migration, invasion, and epithelial-mesenchymal transition in GBM cells (37). CASP1, also known as interleukin-1β converting enzyme, produces IL-1β and mediates the inflammatory response (38). IL-1β secreted by tumor-associated macrophages/microglia has been reported to upregulate other proinflammatory cytokines, promote angiogenesis, and influence almost every step of the glioma progression (39, 40). However, the effects of these genes in necroptosis of glioma cells are still unclear, and our study suggested that they may play roles in glioma.

There were limitations to this study. First, the clinical information obtained from the TCGA and CGGA databases was limited and incomplete. Although our findings might have some clinical value due to the large sample sizes, incorporating detailed information could contribute to a more accurate prognostic evaluation. Second, prognostic information in our cohort was partially missing, and fewer death cases reduced the quality of survival analysis. In future studies, we will continue to follow up with patients and collect survival data. Third, the prognostic model needed additional validation in future large-scale, prospective, multicenter studies. Overall, these findings indicated that necroptosis had a close relationship with immune status and the prognosis of glioma. Necroptosis-related genes may predict prognosis and influence immunotherapeutic effects in glioma patients.

## Data availability statement

The datasets presented in this study can be found in online repositories. The names of the repository/repositories and accession number(s) can be found in the article/**Supplementary Material**.

## Ethics statement

The studies involving human participants were reviewed and approved by Ethics Committee of Guangdong 999 Brain Hospital (Approval number 2021-01-084). Written informed consent to participate in this study was provided by the participants’ legal guardian/next of kin.

## Author contributions

Y-SY and C-MK designed the study. XJ prepared the glioma sections and recorded clinical data. LC and YZ performed bioinformatics analysis. K-WY and W-KL performed immunochemistry staining. J-ML and LC conducted the data analysis. Y-SY conceived the article and wrote the manuscript. S-WC, C-MK and X-ZH confirm the authenticity of all the raw data. All authors contributed to the article and approved the submitted version.

## Funding

The present study was supported by grants from the National Natural Sciences Foundation of China (grant no. 82072336), the Natural Science Fund of Guangdong (grant no. 2019A1515010178, 2019B1515120004, and 2021A1515111125),the Science and Technology Program of Guangzhou (grant nos. 202002020038, 202102010173 and 202103000025), Guangdong basic and Applied Basic Research Foundation project-key project of Regional Joint Fund (2019B1515120004), Dongguan Science and Technology of Social Development Program (201950715023190), Guangzhou Basic and Applied Basic Research project (202102020101), the fellowship of China Postdoctoral Science Foundation (grant no. 2021M700904) and the Project of Administration of Traditional Chinese Medicine of Guangdong Province of China (grant no. 20211180,20202067).

## Conflict of interest

The authors declare that the research was conducted in the absence of any commercial or financial relationships that could be construed as a potential conflict of interest.

## Publisher’s note

All claims expressed in this article are solely those of the authors and do not necessarily represent those of their affiliated organizations, or those of the publisher, the editors and the reviewers. Any product that may be evaluated in this article, or claim that may be made by its manufacturer, is not guaranteed or endorsed by the publisher.
