# Supplementary material for: A novel model based on necroptosis-related genes for predicting immune status and prognosis in glioma
Source: Front Immunol. 2022 Oct 25;13:1027794. doi: 10.3389/fimmu.2022.1027794 (PMC9640834; doi:10.3389/fimmu.2022.1027794)
Supplement: Supplementary file 1 [file DataSheet_1.pdf]

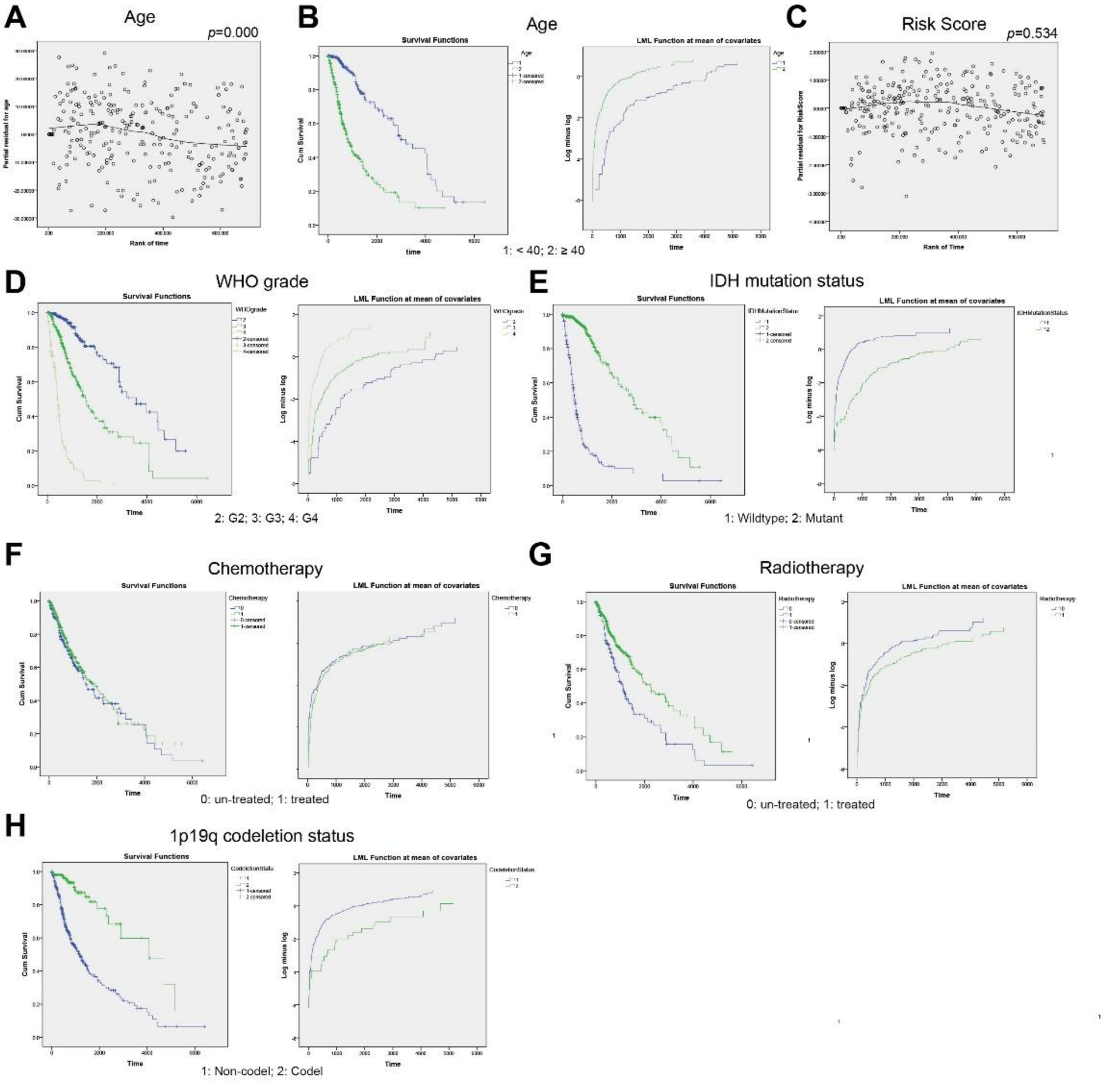

Supplementary Figure 1. Cox proportional hazard (PH) test was conducted before constructing the nomogram. (A) Schoenfeld's test showed that age as a continuous variable did not meet PH test ($p < 0.05$). (B) Age as a categorical variable met the PH test (<40 and ≧ 40). (C) Schoenfeld's test showed that the necroptosis-related risk score met PH test ($p > 0.05$). (D-E) Both WHO grade (G2, G3 and G4) and IDH mutation status (wildtype and mutant) met the PH test. (F) The unparallel and intersecting curves indicated that chemotherapy did not meet PH test. (G-H) Both radiotherapy (un-treated and treated) and 1p19q codeletion status (non-codel and codel) met PH test.
