# Supplementary material for: A novel model based on necroptosis-related genes for predicting immune status and prognosis in glioma
Source: Front Immunol. 2022 Oct 25;13:1027794. doi: 10.3389/fimmu.2022.1027794 (PMC9640834; doi:10.3389/fimmu.2022.1027794)
Supplement: Supplementary file 2 [file DataSheet_2.pdf]

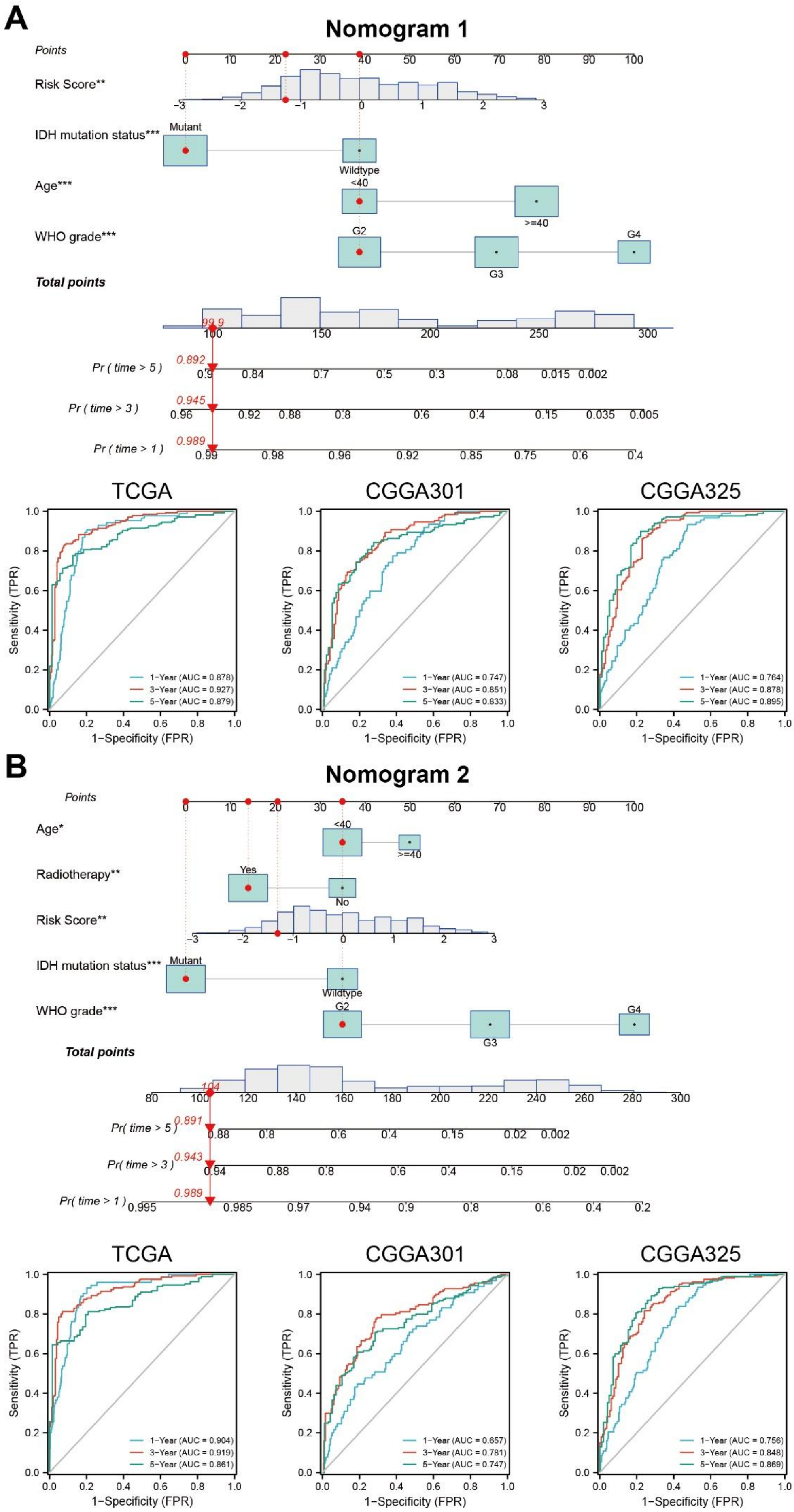
A
Nomogram 1
Points
0 10 20 30 40 50 60 70 80 90 100
Risk Score**
−3 −2 −1 0 1 2 3
IDH mutation status***
Mutant
Wildtype
Age***
<40
>=40
WHO grade***
G2
G3
G4
Total points
99.9
100 150 200 250 300
Pr (time > 5)
0.892
0.9 0.84 0.7 0.5 0.3 0.08 0.015 0.002
Pr (time > 3)
0.945
0.96 0.92 0.88 0.8 0.6 0.4 0.15 0.035 0.005
Pr (time > 1)
0.989
0.99 0.98 0.96 0.92 0.85 0.75 0.6 0.4
TCGA
CGGA301
CGGA325
Sensitivity (TPR)
1−Specificity (FPR)
1−Year (AUC = 0.878)
3−Year (AUC = 0.927)
5−Year (AUC = 0.879)
1−Year (AUC = 0.747)
3−Year (AUC = 0.851)
5−Year (AUC = 0.833)
1−Year (AUC = 0.764)
3−Year (AUC = 0.878)
5−Year (AUC = 0.895)
B
Nomogram 2
Points
0 10 20 30 40 50 60 70 80 90 100
Age*
<40
>=40
Radiotherapy**
Yes
No
Risk Score**
−3 −2 −1 0 1 2 3
IDH mutation status***
Mutant
Wildtype
WHO grade***
G2
G3
G4
Total points
104
80 100 120 140 160 180 200 220 240 260 280 300
Pr(time > 5)
0.891
0.88 0.8 0.6 0.4 0.15 0.02 0.002
Pr(time > 3)
0.943
0.94 0.88 0.8 0.6 0.4 0.15 0.02 0.002
Pr(time > 1)
0.989
0.995 0.985 0.97 0.94 0.9 0.8 0.6 0.4 0.2
TCGA
CGGA301
CGGA325
Sensitivity (TPR)
1−Specificity (FPR)
1−Year (AUC = 0.904)
3−Year (AUC = 0.919)
5−Year (AUC = 0.861)
1−Year (AUC = 0.657)
3−Year (AUC = 0.781)
5−Year (AUC = 0.747)
1−Year (AUC = 0.756)
3−Year (AUC = 0.848)
5−Year (AUC = 0.869)

Supplementary Figure 2. Nomograms and 1-, 3-, and 5-year ROC curves for two models (A) The original model. Age, WHO grade, IDH mutation status and the necroptosis-related risk score were incorporated in the nomogram 1. (B) Since chemotherapy did not meet PH assumption, age, WHO grade, IDH mutation status, radiotherapy and the necroptosis-related risk score were incorporated in the nomogram 2. The AUCs were not improved in three cohorts comparing with nomogram 1.
