# Supplementary material for: A novel model based on necroptosis-related genes for predicting immune status and prognosis in glioma
Source: Front Immunol. 2022 Oct 25;13:1027794. doi: 10.3389/fimmu.2022.1027794 (PMC9640834; doi:10.3389/fimmu.2022.1027794)
Supplement: Supplementary file 3 [file DataSheet_3.pdf]

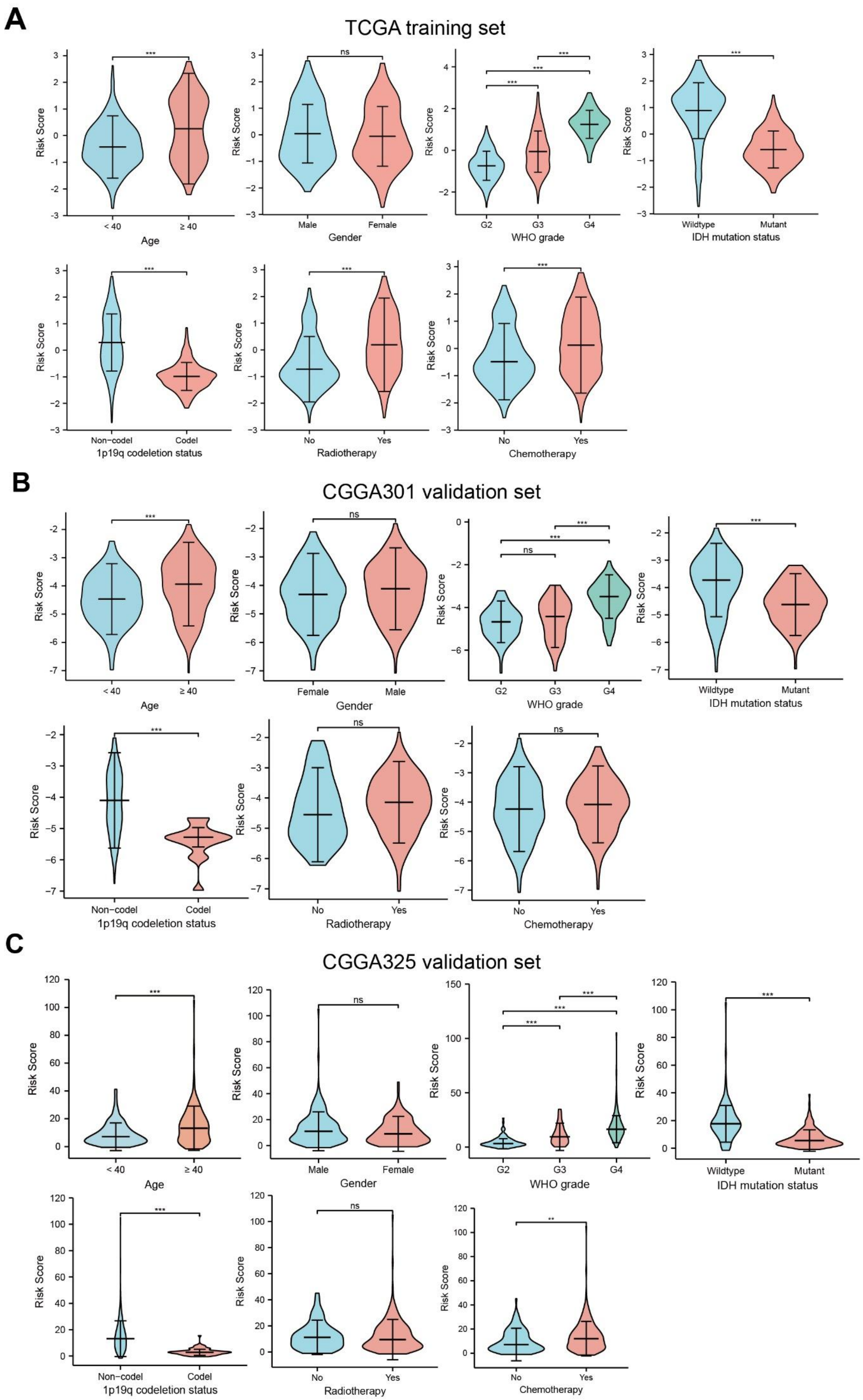
A
TCGA training set
Risk Score
< 40
≥ 40
Age
Male
Female
Gender
G2
G3
G4
WHO grade
Wildtype
Mutant
IDH mutation status
Non−codel
Codel
1p19q codeletion status
No
Yes
Radiotherapy
No
Yes
Chemotherapy
***
ns
B
CGGA301 validation set
Risk Score
< 40
≥ 40
Age
Female
Male
Gender
G2
G3
G4
WHO grade
Wildtype
Mutant
IDH mutation status
Non-codel
Codel
1p19q codeletion status
No
Yes
Radiotherapy
No
Yes
Chemotherapy
***
ns
C
CGGA325 validation set
Risk Score
< 40
≥ 40
Age
Male
Female
Gender
G2
G3
G4
WHO grade
Wildtype
Mutant
IDH mutation status
Non-codel
Codel
1p19q codeletion status
No
Yes
Radiotherapy
No
Yes
Chemotherapy
***
**
ns

Supplementary Figure 3. The associations between the necroptosis-related risk model and clinicopathological features in TCGA training set, CGGA301 and 325 validation sets. (A) The high risk group in TCGA training set was significantly associated with older age (≥ 40), higher WHO grade, IDH wildtype status, and 1p19q non-codeletion, radiotherapy and chemotherapy. The risk scores were not associated with gender. (B) The necroptosis-related risk score in CGGA301 validation set was associated with age, WHO grade, IDH mutation, and 1p19q codeletion status. (C) The risk score in CGGA325 validation set was associated with age, WHO grade, IDH mutation, 1p19q codeletion status and chemotherapy (**$p < 0.01$; ***$p < 0.001$; ns, not significant).
