# Supplementary material for: A novel model based on necroptosis-related genes for predicting immune status and prognosis in glioma
Source: Front Immunol. 2022 Oct 25;13:1027794. doi: 10.3389/fimmu.2022.1027794 (PMC9640834; doi:10.3389/fimmu.2022.1027794)
Supplement: Supplementary file 4 [file DataSheet_4.pdf]

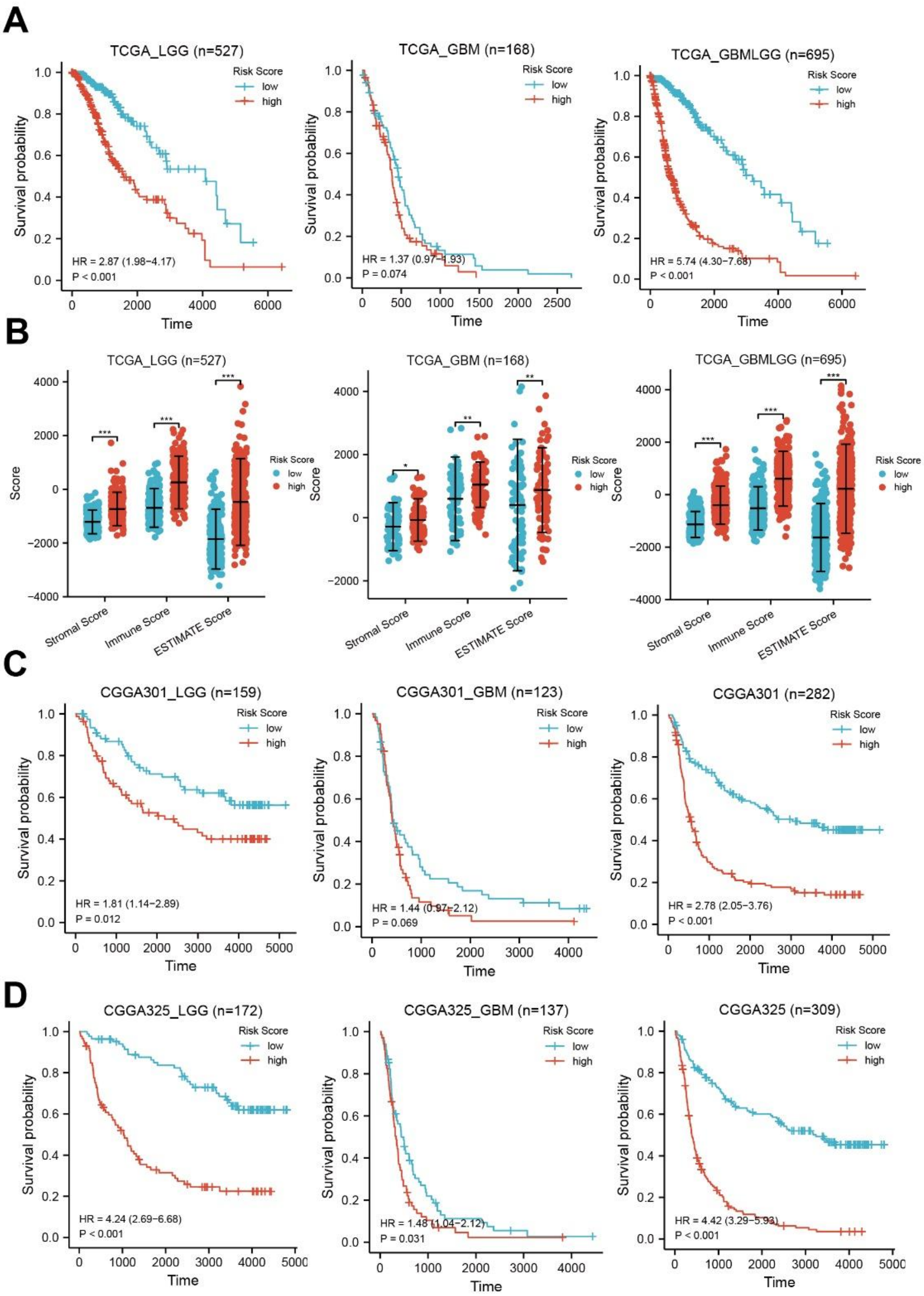

E

The median survival time (days) in each subgroup according to the respective median risk score.

	Low risk group	High risk group
TCGA_LGG	4084 (2660-?)	1578 (1242-2835)
TCGA_GBM	460 (395-548)	382 (323-442)
CGGA301_LGG	—	2190 (1252-?)
CGGA301_GBM	439 (383-823)	412 (372-546)
CGGA325_LGG	—	1028 (679-1394)
CGGA325_GBM	443 (345-681)	333 (277-412)

Supplementary Figure 4. Survival and immune infiltration analysises in each subgroup according to respective median risk score. (A) Patients with high risk score had significantly poor prognosis in TCGA_LGG and TCGA groups. (B) The high risk scores are positively related to immune score, stromal score, and ESTIMAT score based on the ESTIMATE algorithm in three groups of TCGA cohort (*$p < 0.05$; **$p < 0.01$; ***$p < 0.001$). (C) A high risk score correlated with a poor prognosis in CGGA301_LGG, and CGGA301 groups. (D) Similar prediction was found in three groups of CGGA325 cohort. (E) Data are presented as medians and the lower and upper quartiles. The median survival times in CGGA301_LGG and CGGA325_LGG groups are not available since the number of mortality cases was too small to analyze statistically. High risk group had a shorter median survival when compared with low risk group.
