# Supplementary material for: A novel model based on necroptosis-related genes for predicting immune status and prognosis in glioma
Source: Front Immunol. 2022 Oct 25;13:1027794. doi: 10.3389/fimmu.2022.1027794 (PMC9640834; doi:10.3389/fimmu.2022.1027794)
Supplement: Supplementary file 5 [file DataSheet_5.pdf]

A

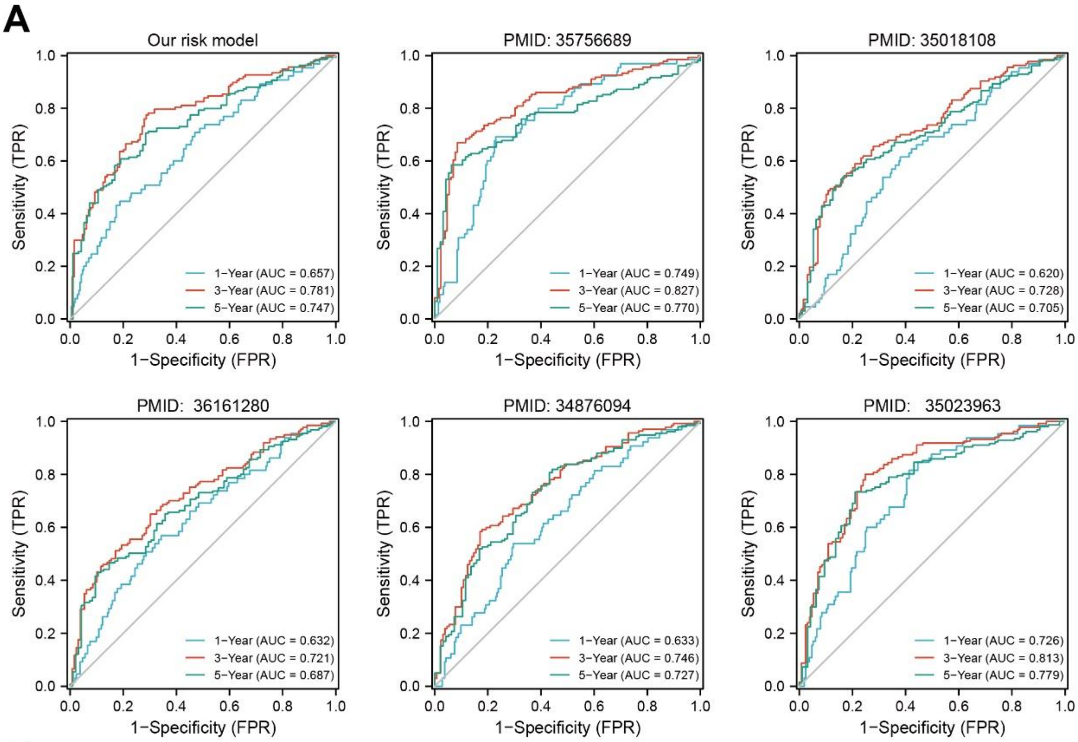

B

References (PMID)	Number of genes in the model	1-, 3-, 5-year AUCs in CGGA301 cohort	C-index of the model	Validation in clinical samples
35756689(1)	7	0.749, 0.827, 0.770	0.697 (0.681-0.714)	No
35018108(2)	5	0.620, 0.728, 0.705	0.630 (0.611-0.650)	Yes (12 pairs, gene expression)
36161280(3)	3	0.632, 0.721, 0.687	0.624 (0.603-0.645)	Yes (12 pairs, gene expression)
34876094(4)	10	0.633, 0.746, 0.727	0.645 (0.625-0.666)	No
35023963(5)	9	0.726, 0.813, 0.779	0.687 (0.670-0.705)	No
Our risk model	3	0.657, 0.781, 0.747	0.668 (0.648-0.688)	Yes (43 paired samples, gene expression and risk model)

Supplementary Figure 5. Benchmarking prognostic models. (A) ROC curves of partially existing models and the model proposed in this study based on CGGA301 cohort. (B) AUCs and C-index showed that our three-gene prognostic model achieved similarly great prediction performance compared with existing models.

References

1. Yun D, Wang X, Wang W, Ren X, Li J, Wang X, et al. A Novel Prognostic Signature Based on Glioma Essential Ferroptosis-Related Genes Predicts Clinical Outcomes and Indicates Treatment in Glioma. Front Oncol. 2022;12:897702.
2. Zheng J, Zhou Z, Qiu Y, Wang M, Yu H, Wu Z, et al. A Pyroptosis-Related Gene Prognostic Index Correlated with Survival and Immune Microenvironment in Glioma. J Inflamm Res.

2022;15:17-32.
3. Zhang M, Cheng Y, Xue Z, Sun Q, Zhang J. A novel pyroptosis-related gene signature predicts the prognosis of glioma through immune infiltration. BMC Cancer. 2021;21(1):1311.
4. Chen J, Chen S, Li B, Zhou S, Lin H. A pyroptosis-related signature predicts prognosis and indicates immune microenvironment infiltration in glioma. Cancer Med. 2022.
5. Sun W, Yan J, Ma H, Wu J, Zhang Y. Autophagy-Dependent Ferroptosis-Related Signature is Closely Associated with the Prognosis and Tumor Immune Escape of Patients with Glioma. Int J Gen Med. 2022;15:253-70.
