# Supplementary material for: A novel model based on necroptosis-related genes for predicting immune status and prognosis in glioma
Source: Front Immunol. 2022 Oct 25;13:1027794. doi: 10.3389/fimmu.2022.1027794 (PMC9640834; doi:10.3389/fimmu.2022.1027794)
Supplement: Supplementary file 6 [file DataSheet_6.pdf]

CGGA301

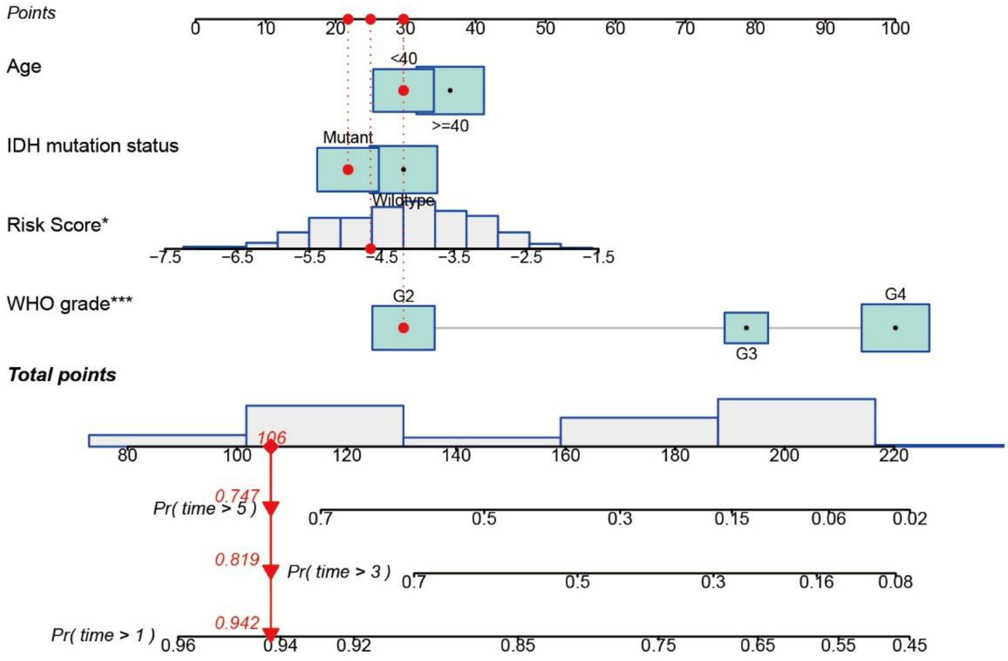

CGGA325

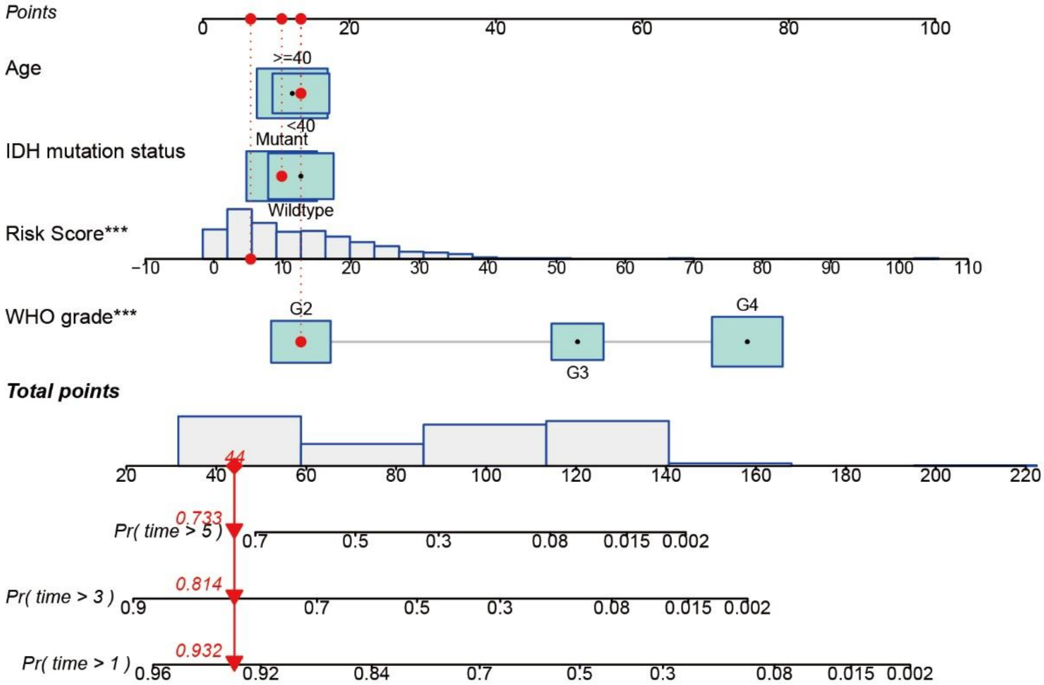

Supplementary Figure 6. Prognostic nomogram to predict the 1-, 3-, and 5-year OS in CGGA301 and 325 validation sets. Red dots represent one case from the cohort.
