# Supplementary material for: A novel model based on necroptosis-related genes for predicting immune status and prognosis in glioma
Source: Front Immunol. 2022 Oct 25;13:1027794. doi: 10.3389/fimmu.2022.1027794 (PMC9640834; doi:10.3389/fimmu.2022.1027794)
Supplement: Supplementary file 7 [file Table_1.docx]

**Supplementary Table 1. The clinical characteristics of glioma patients in TCGA training set, and CGGA301, 325 validation sets.**

| **Characteristics** | **TCGA cohort** | **CGGA301 cohort** | **CGGA325 cohort** |
| --- | --- | --- | --- |
| n | 695 | 285 | 313 |
| **Age, n (%)** |  |  |  |
| < 40 | 266 (38.3%) | 126 (44.2%) | 123 (39.3%) |
| ≥ 40 | 429 (61.7%) | 157 (55.1%) | 190 (60.7%) |
| NA | 0 | 2 (0.7%) | 0 |
| **Sex, n (%)** |  |  |  |
| Female | 297 (42.7%) | 117 (41.1%) | 116 (37.1%) |
| Male | 398 (57.3%) | 168 (58.9%) | 197 (62.9%) |
| **WHO grade, n (%)** |  |  |  |
| G2 | 259 (37.3%) | 106 (37.2%) | 98 (31.3%) |
| G3 | 268 (38.6%) | 53 (18.6%) | 74 (23.6%) |
| G4 | 152 (21.9%) | 123 (43.2%) | 137 (43.8%) |
| NA | 16 (2.2%) | 3 (1.0%) | 4 (1.3%) |
| **IDH mutation status,**  **n (%)** |  |  |  |
| Wildtype | 250 (36.0%) | 157 (55.1%) | 145 (46.3%) |
| Mutant | 421 (60.6%) | 127 (44.6%) | 167 (53.4%) |
| NA | 24 (3.4%) | 1 (0.3%) | 1 (0.3%) |
| **1p19q codeletion status,**  **n (%)** |  |  |  |
| Non-codel | 489 (70.4%) | 73 (25.6%) | 243 (77.6%) |
| Codel | 167 (24.0%) | 16 (5.6%) | 62 (19.8%) |
| NA | 39 (5.6%) | 196 (68.8%) | 8 (2.6%) |
| **Radiotherapy, n (%)** |  |  |  |
| No | 192 (27.6%) | 39 (13.7%) | 62 (19.8%) |
| Yes | 424 (61.0%) | 234 (82.1%) | 241 (77.0%) |
| NA | 79 (11.4%) | 12 (4.2%) | 10 (3.2%) |
| **Chemotherapy, n (%)** |  |  |  |
| No | 285 (41.0%) | 141 (49.5%) | 110 (35.1%) |
| Yes | 407 (58.6%) | 133 (46.7%) | 190 (60.7%) |
| NA | 3 (0.4%) | 11 (3.8%) | 13 (4.2%) |
| **Survival status, n (%)** |  |  |  |
| Alive | 423 (60.9%) | 100 (35.1%) | 95 (30.4%) |
| Dead | 272 (39.1%) | 185 (64.9%) | 218 (69.6%) |
| **Follow-up time,**  **median (IQR)** | 569 (307, 1109) | 812 (363, 3107) | 705 (289, 2568) |
