# Supplementary material for: A novel model based on necroptosis-related genes for predicting immune status and prognosis in glioma
Source: Front Immunol. 2022 Oct 25;13:1027794. doi: 10.3389/fimmu.2022.1027794 (PMC9640834; doi:10.3389/fimmu.2022.1027794)
Supplement: Supplementary file 8 [file Table_2.docx]

**Supplementary Table 2. The clinical characteristics of glioma patients in our third cohort.**

| **Characteristics** | **Our third cohort** |
| --- | --- |
| n | 43 |
| **Age, median (IQR)** | 34 (22, 47) |
| **Gender, n (%)** |  |
| Female | 20 (46.5%) |
| Male | 23 (53.5%) |
| **Grade, n (%)** |  |
| LGG | 13 (30.2%) |
| HGG | 12 (27.9%) |
| NA | 18 (41.9%) |
| **IDH mutation status, n (%)** |  |
| Wildtype | 16 (37.2%) |
| Mutant | 18 (41.9%) |
| NA | 9 (20.9%) |
| **1p19q codeletion status, n (%)** |  |
| Non-codel | 8 (18.6%) |
| Codel | 10 (23.3%) |
| NA | 25 (58.1%) |
| **MGMT methylation** |  |
| Negative | 13 (30.2%) |
| Positive | 10 (23.3%) |
| NA | 20 (46.5%) |
| **Postoperative recurrence** |  |
| No | 8 (18.6%) |
| Yes | 13 (30.2%) |
| NA | 22 (51.2%) |
| **Survival status, n (%)** |  |
| Alive | 29 (67.4%) |
| Dead | 2 (4.7%) |
| NA | 12 (27.9%) |
| **Follow-up time, median (IQR)** | 1 098 (491, 1824) |
