# Supplementary material for: A novel model based on necroptosis-related genes for predicting immune status and prognosis in glioma
Source: Front Immunol. 2022 Oct 25;13:1027794. doi: 10.3389/fimmu.2022.1027794 (PMC9640834; doi:10.3389/fimmu.2022.1027794)
Supplement: Supplementary file 10 [file Table_4.docx]

**Supplementary Table 4.1 Multivariate Cox regression analyses in TCGA training set.**

| Characteristics | Total(N) | HR(95% CI) Univariate analysis | *p* value |  |
| --- | --- | --- | --- | --- |
| **Sex** | 695 |  | 0.062 | |
| Female | 297 | Reference |  | |
| Male | 398 | 1.262 (0.988-1.610) | 0.062 | |
| **Age** | 695 |  | <0.001 | |
| < 40 | 266 | Reference |  | |
| ≥ 40 | 429 | 4.304 (3.172-5.842) | <0.001 | |
| **WHO grade** | 679 |  | <0.001 | |
| G2 | 259 | Reference |  | |
| G3 | 268 | 3.208 (2.216-4.645) | <0.001 | |
| G4 | 152 | 18.213 (12.443-26.659) | <0.001 | |
| **IDH mutation status** | 671 |  | <0.001 | |
| Wildtype | 250 | Reference |  | |
| Mutant | 421 | 0.137 (0.105-0.180) | <0.001 | |
| **1p19q codeletion status** | 656 |  | <0.001 | |
| Non-codel | 489 | Reference |  | |
| Codel | 167 | 0.220 (0.141-0.345) | <0.001 | |
| **Chemotherapy** | 692 |  | 0.008 | |
| No | 285 | Reference |  | |
| Yes | 407 | 1.454 (1.104-1.913) | 0.008 | |
| **Radiotherapy** | 616 |  | <0.001 | |
| No | 192 | Reference |  | |
| Yes | 424 | 2.114 (1.515-2.950) | <0.001 | |
| **Risk Score** | 695 | 2.718 (2.397-3.083) | <0.001 | |

Sex was not associated with the prognosis of glioma and excluded in the multivariate Cox regression.

**Supplementary Table 4.2 Multivariate Cox regression analyses in TCGA training set.**

| Characteristics | Total(N) | HR(95% CI) Multivariate analysis | *p* value |
| --- | --- | --- | --- |
| **Age** | 695 |  |  |
| < 40 | 266 |  |  |
| ≥ 40 | 429 | 2.133 (1.409-3.229) | <0.001 |
| **WHO grade** | 679 |  |  |
| G2 | 259 |  |  |
| G3 | 268 | 2.402 (1.464-3.942) | <0.001 |
| G4 | 152 | 5.109 (2.766-9.437) | <0.001 |
| **IDH mutation status** | 671 |  |  |
| Wildtype | 250 |  |  |
| Mutant | 421 | 0.375 (0.234-0.600) | <0.001 |
| **1p19q codeletion status** | 656 |  |  |
| Non-codel | 489 |  |  |
| Codel | 167 | 0.584 (0.324-1.051) | 0.073 |
| **Chemotherapy** | 692 |  |  |
| No | 285 |  |  |
| Yes | 407 | 0.690 (0.487-0.978) | 0.037 |
| **Radiotherapy** | 616 |  |  |
| No | 192 |  |  |
| Yes | 424 | 0.645 (0.427-0.975) | 0.037 |
| **RiskScore** | 695 | 1.333 (1.071-1.658) | **0.010** |

Multivariate Cox regression showed that age, WHO grade, IDH mutation status, chemotherapy, radiotherapy and risk score were independent prognostic factors in TCGA training set.

**Supplementary Table 4.3 Univariate and multivariate Cox regression analyses in CGGA301 validation set.**

| Characteristics | Total(N) | | HR(95% CI) Univariate analysis | *p* value | HR(95% CI)  Multivariate analysis | *p* value |
| --- | --- | --- | --- | --- | --- | --- |
| **Age** | | 283 |  | <0.001 |  |  |
| < 40 | | 126 | Reference |  |  |  |
| ≥ 40 | | 157 | 1.878 (1.393-2.531) | <0.001 | 1.027 (0.741-1.423) | 0.874 |
| **WHO grade** | | 282 |  | <0.001 |  |  |
| G2 | | 106 | Reference |  |  |  |
| G3 | | 53 | 2.990 (1.892-4.725) | <0.001 | 3.047 (1.904-4.877) | <0.001 |
| G4 | | 123 | 6.938 (4.725-10.187) | <0.001 | 4.781 (3.024-7.559) | <0.001 |
| **IDH mutation status** | | 284 |  | <0.001 |  |  |
| Wildtype | | 157 | Reference |  |  |  |
| Mutant | | 127 | 0.391 (0.287-0.533) | <0.001 | 0.819 (0.570-1.176) | 0.279 |
| **Chemotherapy** | | 274 |  | 0.429 |  |  |
| No | | 141 | Reference |  |  |  |
| Yes | | 133 | 1.126 (0.839-1.513) | 0.429 |  |  |
| **Radiotherapy** | | 273 |  | 0.005 |  |  |
| No | | 39 | Reference |  |  |  |
| Yes | | 234 | 0.569 (0.384-0.844) | 0.005 | 0.491 (0.327-0.738) | <0.001 |
| **Risk Score** | | 285 | 1.852 (1.575-2.178) | <0.001 | 1.289 (1.072-1.552) | **0.007** |

1p19q codeletion status was not included in the analysis due to excessive missing data. The necroptosis-related risk score could serve as an independent prognostic factor in CGGA301 validation set.

**Supplementary Table 4.4 Univariate and multivariate Cox regression analyses in CGGA325 validation set.**

| Characteristics | Total(N) | HR(95% CI) Univariate analysis | *p* value | HR(95% CI)  Multivariate analysis | *p* value |
| --- | --- | --- | --- | --- | --- |
| **Age** | 313 |  | <0.001 |  |  |
| < 40 | 123 | Reference |  |  |  |
| ≥ 40 | 190 | 1.638 (1.236-2.173) | <0.001 | 1.052 (0.764-1.450) | 0.754 |
| **WHO grade** | 309 |  | <0.001 |  |  |
| G2 | 98 | Reference |  |  |  |
| G3 | 74 | 3.498 (2.287-5.348) | <0.001 | 3.693 (2.318-5.884) | <0.001 |
| G4 | 137 | 8.902 (5.996-13.215) | <0.001 | 6.164 (3.812-9.968) | <0.001 |
| **IDH mutation status** | 312 |  | <0.001 |  |  |
| Wildtype | 145 | Reference |  |  |  |
| Mutant | 167 | 0.354 (0.269-0.468) | <0.001 | 1.060 (0.741-1.515) | 0.751 |
| **1p19q codeletion status** | 305 |  | <0.001 |  |  |
| Non-codel | 243 | Reference |  |  |  |
| Codel | 62 | 0.170 (0.104-0.277) | <0.001 | 0.336 (0.196-0.576) | <0.001 |
| **Chemotherapy** | 300 |  | 0.014 |  |  |
| No | 110 | Reference |  |  |  |
| Yes | 190 | 1.445 (1.078-1.938) | 0.014 | 0.648 (0.466-0.903) | 0.010 |
| **Radiotherapy** | 303 |  | 0.005 |  |  |
| No | 62 | Reference |  |  |  |
| Yes | 241 | 0.631 (0.457-0.872) | 0.005 | 0.801 (0.568-1.129) | 0.204 |
| **Risk Score** | 313 | 1.046 (1.038-1.053) | <0.001 | 1.025 (1.013-1.037) | **<0.001** |

The necroptosis-related risk score could serve as an independent prognostic factor in CGGA325 validation set.

However, chemotherapy did not meet cox proportional hazard test (Supplementary Figure 1). Age, WHO grade, IDH mutation status, and risk score were integrated into the nomogram model. Since the AUCs were not improved in TCGA, CGGA301 and CGGA325 cohorts when radiotherapy was included in the nomogram (Supplementary Figure 2).
