# Supplementary material for: A novel model based on necroptosis-related genes for predicting immune status and prognosis in glioma
Source: Front Immunol. 2022 Oct 25;13:1027794. doi: 10.3389/fimmu.2022.1027794 (PMC9640834; doi:10.3389/fimmu.2022.1027794)
Supplement: Supplementary file 13 [file Table_7.docx]

**Supplementary Table 7. Clinicopathologic characteristics of glioma patients in the TCGA cohort based on treatment received.**

| Characteristic | Non-  radiotherapy | Radiotherapy | *p* value | Non- chemotherapy | Chemotherapy | *p* value |
| --- | --- | --- | --- | --- | --- | --- |
| n | 192 | 424 |  | 285 | 407 |  |
| **Age, n (%)** |  |  | 0.001 |  |  | 0.027 |
| < 40 | 91 (14.8%) | 141 (22.9%) |  | 124 (17.9%) | 142 (20.5%) |  |
| ≥ 40 | 101 (16.4%) | **283 (45.9%)** |  | 161 (23.3%) | **265 (38.3%)** |  |
| **WHO grade,**  **n (%)** |  |  | < 0.001 |  |  | < 0.001 |
| G2 | 130 (21.6%) | 96 (16%) |  | 169 (25%) | 89 (13.1%) |  |
| G3 | 41 (6.8%) | **191 (31.8%)** |  | 68 (10%) | **199 (29.4%)** |  |
| G4 | 21 (3.5%) | **122 (20.3%)** |  | 46 (6.8%) | **106 (15.7%)** |  |
| **IDH mutation status, n (%)** |  |  | < 0.001 |  |  | < 0.001 |
| Wildtype | 44 (7.4%) | 178 (30%) |  | 82 (12.3%) | 168 (25.1%) |  |
| Mutant | 147 (24.7%) | 225 (37.9%) |  | 198 (29.6%) | 221 (33%) |  |
| **1p19q codeletion status, n (%)** |  |  | < 0.001 |  |  | 0.001 |
| Non-codel | 103 (17.7%) | **330 (56.8%)** |  | 186 (28.4%) | **302 (46.2%)** |  |
| Codel | 84 (14.5%) | 64 (11%) |  | 88 (13.5%) | 78 (11.9%) |  |
| **Survival status, n (%)** |  |  | < 0.001 |  |  | < 0.001 |
| Alive | 150 (24.4%) | 221 (35.9%) |  | 215 (31.1%) | 206 (29.8%) |  |
| Dead | 42 (6.8%) | 203 (33%) |  | 70 (10.1%) | 201 (29%) |  |
| **Follow-up time,**  **Median (IQR)** | 655.5  (204.5, 1205.5) | 576  (384.75, 1063.75) | 0.599 | 497  (114, 993) | 604  (406.5, 1186) | < 0.001 |
| **RiskScore, median (IQR)** | -0.73  (-1.15, 0.08) | 0.19  (-0.62, 1.13) | < 0.001 | -0.49  (-1.04, 0.37) | 0.12  (-0.72, 1.04) | < 0.001 |

In the TCGA cohort, patients receiving chemoradiotherapy or radiotherapy were mostly older than 40 years, with high WHO grade and 1p19q codeletion status, which were significantly associated with high risk score.
