# Supplementary material for: A novel model based on necroptosis-related genes for predicting immune status and prognosis in glioma
Source: Front Immunol. 2022 Oct 25;13:1027794. doi: 10.3389/fimmu.2022.1027794 (PMC9640834; doi:10.3389/fimmu.2022.1027794)
Supplement: Supplementary file 14 [file Table_8.docx]

**Supplementary Table 8. C-index of the independent prognostic factors in TCGA training set, CGGA301 and CGGA325 validation sets.**

| **Characteristics** | **TCGA cohort** | **CGGA301 cohort** | **CGGA325 cohort** |
| --- | --- | --- | --- |
| **Age** | 0.672(0.661-0.684) | 0.587(0.568-0.605) | 0.551(0.533-0.569) |
| **WHO grade** | 0.786 (0.773-0.799) | 0.717 (0.702-0.732) | 0.721 (0.707-0.735) |
| **IDH mutation status** | 0.775 (0.763-0.787) | 0.616 (0.598-0.635) | 0.620 (0.603-0.637) |
| **Risk Score** | 0.788 (0.774-0.803) | 0.666 (0.646-0.687) | 0.727 (0.710-0.743) |
| **Nomogram** | 0.843(0.832-0.855) | 0.724(0.708-0.741) | 0.739(0.723-0.755) |
